# Tuning Nanopore Diameter of Titanium Surfaces to Improve Human Gingival Fibroblast Response

**DOI:** 10.3390/ijms19102881

**Published:** 2018-09-22

**Authors:** Maria del Mar Ferrà-Cañellas, Maria Antonia Llopis-Grimalt, Marta Monjo, Joana Maria Ramis

**Affiliations:** 1 Group of Cell Therapy and Tissue Engineering, Research Institute on Health Sciences (IUNICS), University of the Balearic Islands. Ctra. Valldemossa km 7.5, 07122 Palma de Mallorca, Spain; mar.ferra@uib.cat (M.d.M.F.-C.); mantonia.llopis@uib.es (M.A.L.-G.); 2 Balearic Islands Health Research Institute (IdISBa), 07010 Palma de Mallorca, Spain

**Keywords:** nanopore diameter, electrochemical anodization, soft tissue integration, surface area

## Abstract

The aim of this study was to determine the optimal nanopore diameter of titanium nanostructured surfaces to improve human gingival fibroblast (hGF) response, with the purpose of promoting gingiva integration to dental implant abutments. Two TiO_2_ nanoporous groups with different diameters (NP-S ~48 nm and NP-B ~74 nm) were grown on Ti foils using an organic electrolyte containing fluoride by electrochemical oxidation, varying the applied voltage and the interelectrode spacing. The surfaces were characterized by scanning electron microscope (SEM), atomic force microscopy (AFM), and contact angle. The hGF were cultured onto the different surfaces, and metabolic activity, cytotoxicity, cell adhesion, and gene expression were analyzed. Bigger porous diameters (NP-B) were obtained by increasing the voltage used during anodization. To obtain the smallest diameter (NP-S), apart from lowering the voltage, a lower interelectrode spacing was needed. The greatest surface area and number of peaks was found for NP-B, despite these samples not being the roughest as defined by R_a_. NP-B had a better cellular response compared to NP-S. However, these effects had a significant dependence on the cell donor. In conclusion, nanoporous groups with a diameter in the range of 74 nm induce a better hGF response, which may be beneficial for an effective soft tissue integration around the implant.

## 1. Introduction

The main challenge in developing a new generation of dental implants is to combine an improved osseointegration with a greater soft tissue integration around the implant, which will promote the durability of the implant [1,2]. An effective soft tissue barrier, with gingival tissue attached to the implant abutment, may improve protective functions, preventing periodontal disease (inflammation of the supporting tissues of the teeth with progressive attachment loss and bone destruction) [3,4,5,6].

To enhance soft tissue compatibility, researchers try to manipulate the implant surface structure at the nanolevel [7], attempting to mimic the dimensions and properties of the physiological extracellular matrix [8,9]. Current nanostructuration methods allow the formation of biocompatible surfaces and the production of distinct morphologies, for instance, by changing the electrochemical conditions when using electrochemical anodization—one of the methods most frequently used [10].

Cell behavior on biomaterial surfaces depends fully on its biocompatibility and surface properties [2,11]. Interaction between cells and nanostructures provides the possibility of controlling the cell culture in order to achieve the desired biological responses [8,9,12]. Modulation of cell behavior through physicochemical surface modification may be mediated by a phenomenon called mechanotransduction, the conversion of mechanical signals into biochemical signals [13,14]. In fact, the cell fate of mesenchymal stem cells can be modulated by material interaction through nanosize integrin receptors [14].

Here, we wanted to determine the optimal nanoporous diameter of Ti nanostructured surfaces that exhibits a beneficial effect on fibroblast cell culture. For this purpose, human gingival fibroblasts (hGF) culture assays were carried out to analyze the effects of nanoporous diameters on cytotoxicity, cell adhesion, metabolic activity, and gene expression of genes related to the synthesis and organization of the extracellular matrix and collagen deposition.

## 2. Results and Discussion

### 2.1. Surface Characterization

Anodic oxidation is generally a simple, versatile, and low-cost technique to produce nanostructures on the surface of Ti [9]. To achieve highly ordered porous systems, it is crucial to use optimized anodization parameters, such as anodization time, temperature and potential, as well as electrolyte composition and properties (pH, conductivity, or viscosity) [9,15,16]. The topographical features of the obtained structure are strongly affected by these parameters, and they can therefore be modified to obtain the desired nanostructure.

As shown in the supporting information (Table S1), changes in parameters such as the electrolyte, voltage, or time changed the obtained nanostructure, and prove the importance of using the right parameters to achieve the desired well-ordered nanostructure. These results also show the difficulties encountered with anodization assays in obtaining a regular and defined nanostructure, since there are many factors that could alter the assay, including room temperature or humidity. After the optimization, two anodizing conditions were selected for the present study.

**Figure 1:**
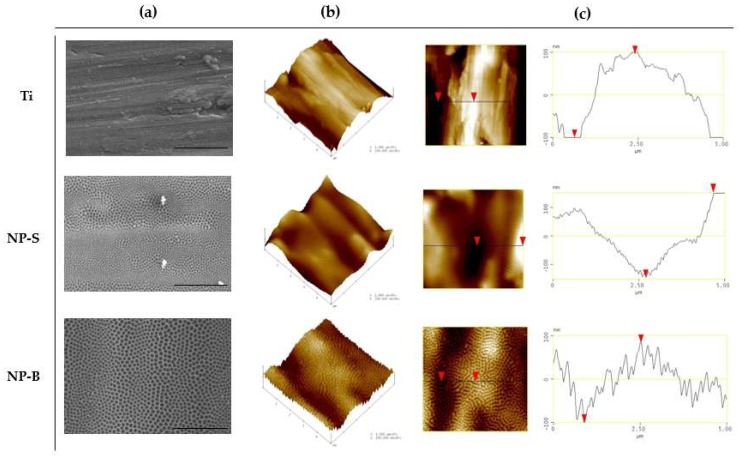
Surface characterization by scanning electron microscope (SEM) and atomic force microscopy (AFM) images of nanoporous arrays (NP-S, NP-B) and the Ti control surface. (**a**) SEM images of the surfaces (scale bar = 2 μm). (**b**) Three-dimensional reconstructions based on 5 µm × 5 µm scans obtained by AFM. (**c**) Two-dimensional images and cross-sectional profiles obtained by AFM. Red triangle represented the highest and lowest position of the surface.

As shown by the scanning electron microscope (SEM) images (Figure 1), two different groups of well-aligned porous structures were obtained by changing the anodization voltage and electrode interspace (NP-S and NP-B). Bigger pore size was obtained when using a higher voltage and electrode interspace, as shown in Table 1. Our results are in agreement with previous reports, showing that the diameter of the TiO_2_ nanotubes increases linearly with increasing anodizing voltage [17,18,19,20].

Nevertheless, according to our experience, in order to obtain a different porous diameter it was not enough to change only the voltage, it was also necessary to modify the interelectrode spacing; thus, demonstrating the important role of this parameter, which particularly affects the electrolyte conductivity and concentration during anodization, especially in organic electrolytes [21].

It is worth mentioning the importance of using a pre-anodized (“aged”) electrolyte. Some studies suggest that the electrolyte needs an aging process before being used in the anodization [20]. Aging the electrolyte is another anodization factor with importance in improving the quality of the imprint pattern, and in obtaining a defined initiation of the tube growth [22].

The topographical features of the obtained nanostructured surfaces were evaluated by atomic force microscopy (AFM) and wettability by contact angle measurements, as shown in Table 1 and Figure 1. In order to describe roughness, parameters such as average surface roughness (R_a_), root mean square surface roughness (R_q_), and maximum surface roughness (R_max_) are commonly used; however, these parameters might be insufficient for the description of the nanoarchitecture of surfaces, as they give no indication of the shape or spatial density of peaks [23]. For this reason, additional horizontal parameters are used: Skewness (R_skw_), kurtosis (R_kur_), surface area difference (R_sa_), and peak counts (R_pc_) [23].

From the topographical parameters evaluated, no significant differences were found for R_a_, R_sk_, and R_kur_. In contrast, R_sa_ and R_pc_ values increased, as did pore diameter. The greatest surface area and number of peaks was found for NP-B, being significantly different to the control and NP-S surfaces. In accordance with the roughness parameters, while visual inspection of AFM scans revealed similar surface features and good agreement with the SEM images, cross-sectional profiles revealed a great difference between the NP-B surface compared to the Ti control and NP-S surfaces. R_sa_ is defined as the percentage increase of the three-dimensional surface area over the two-dimensional surface area, which accounts for both the magnitude and the frequency of surface features, and provides a good measure of surface roughness [24]. In our study, the greatest surface area and number of peaks was found for the NP-B surface, despite these samples not being the roughest as defined by R_a_. This difference between R_a_ and R_sa_ when assessing the roughness is explained by the dependence on the frequency and distribution of surface projections; while an increase in peak count may not significantly affect the average roughness, it can represent an increase of surface area difference [24].

These topographical changes at the nanolevel rebound at surface wettability. Results for water contact angle (Table 1) indicated that the initial hydrophilic surface of Ti samples was changed by nanostructures, but they still retained a hydrophilic character (<90°). For NP-S, we obtained the highest contact angle surface compared to the Ti control and to the NP-B group, thus making it the most hydrophobic of the studied surfaces [25]. After anodic modification, NP-B surfaces showed the same hydrophilic character as the Ti control, pointing to the importance of pore size in the wettability of the surface. Bigger pore size could lead to the most favorable surface in relation to hydrophilicity, which is in accordance with previous studies, where fibroblasts show greater attachment and spreading on hydrophilic surfaces compared to hydrophobic ones [11].

### 2.2. Cell Response to Nanoporous Surfaces

Abutment soft tissue integration is conditioned by the adhesion and spreading ability on implant surfaces of its principal cells—fibroblasts [11]. Fibroblasts are of mesenchymal origin and play a major role in the development, maintenance, and repair of gingival connective tissues. The principal function of fibroblasts is to synthesize and maintain the components of the extracellular matrix of the connective tissue [26]. It is generally agreed that cell and tissue responses are sensitive to the implant surface chemical and physical features [1,27,28]. Thus, a combination of an optimal surface and the mechanical properties of titanium could lead to successful dental implants [12].

**Figure 2:**
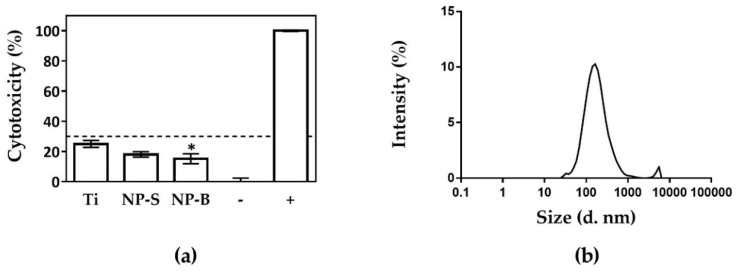
Analysis of cytotoxicity of human gingival fibroblast cells seeded on Ti and TiO_2_ nanoporous surfaces. (**a**) Lactate dehydrogenase (LDH) activity measured from culture media of hGF cells seeded on different surfaces after 48 h, was measured for evaluation of cytotoxicity. The positive control (100%) was cell culture media from cells seeded onto tissue culture plastic and incubated with 1% Triton X-100. The negative control (0%) was cell culture media from cells seeded on tissue culture plastic without any treatment. Mean ± S.E.M (*n* = 12) are represented. Differences between groups were assessed by ANOVA and post-hoc LSD test: * *p* < 0.05 versus Ti. (**b**) Particle-size distribution (nm) versus percentage of particle intensity determined by dynamic light scattering (DLS) of NP-B nanoparticle test release.

In order to test the cell response to the different surfaces of the study, first, cell cytotoxicity was analyzed. Figure 2a shows that all surfaces gave cytotoxicity values lower than the 30% limit established for medical implants according to ISO-10993:5 [29]. Here, it is demonstrated anodization treatment was safe and nontoxic for cells, indicating that electrochemical oxidation of Ti sheets did not change the excellent biocompatibility of Ti control surfaces on primary hGF, one of the most important factors for selecting dental abutment materials [6,30]. Previous studies have shown that tube diameters of approximately 100 nm induce programmed cell death [15,31]. Hence, our results manifest the importance of nanostructure diameters on cell cytotoxicity.

We also evaluated the release of nanoparticles from the developed surfaces. Curiously, only the NP-B group, the more biocompatible of the tested surfaces, showed nanoparticle release, exhibiting two peaks between 50 and 1000 nm (Figure 2b), with a mean size of 568.3 nm and a concentration of 2.85 × 10^8^ particles/mL. These results disagree with previous results from our group [32], where NP surfaces obtained on Ti disks were toxic for the cells due to high nanoparticle release, with their levels being 1.08 × 10^9^ particles/mL. It is to be highlighted that the method for obtaining those structures was different from the one used here; for instance, Ti disks of 2 mm in height were used instead of the Ti foils of 0.127 mm in height that are used here, and the electrolyte was not aged in contrast to the one used for the present work. Our results underline the importance of all the methodological details for nanostructure fabrication on the final results achieved. In fact, the use of two-step procedures in anodization methods might also lead to the presence of impurities [2], which can be harmful for the cultured cells [19,33].

Increased bone cell functions may rely on the degree of the nanostructured surface roughness [9]. It has been hypothesized that osteoblasts recognize different surface roughness through the interaction of proteins in the extracellular matrix [27]. In the same way, studies suggested that the addition of rough surface features may increase connective tissue attachment [34], and that it is required for the formation of a stable soft tissue seal around the implant abutments [35,36]. Moreover, fibroblasts are sensitive to changes in surface roughness and hydrophilicity too [37]. Such events are possibly mediated by nanotopography-induced mechanotransduction pathways. Nanostructuring of appropriate size and arrangement may provide the necessary physical cues that cell receptors require to organize the cytoskeleton, and to propagate mechanical signals towards the nucleus, modulating the cell fate [14]. It is suggested that the mechanisms of mechanotransduction could play a role in the relation between adhesion and osteogenic differentiation [13].

Therefore, due to their highly defined geometry combined with a large surface area, it was expected that NP-B presented better cell interactions than the smallest nanoporous NP-S. Previous works on size-dependent cell interactions has shown that mesenchymal stem cells (MSCs) respond in a pronounced way to the diameter of nanotubes [31].

**Figure 3:**
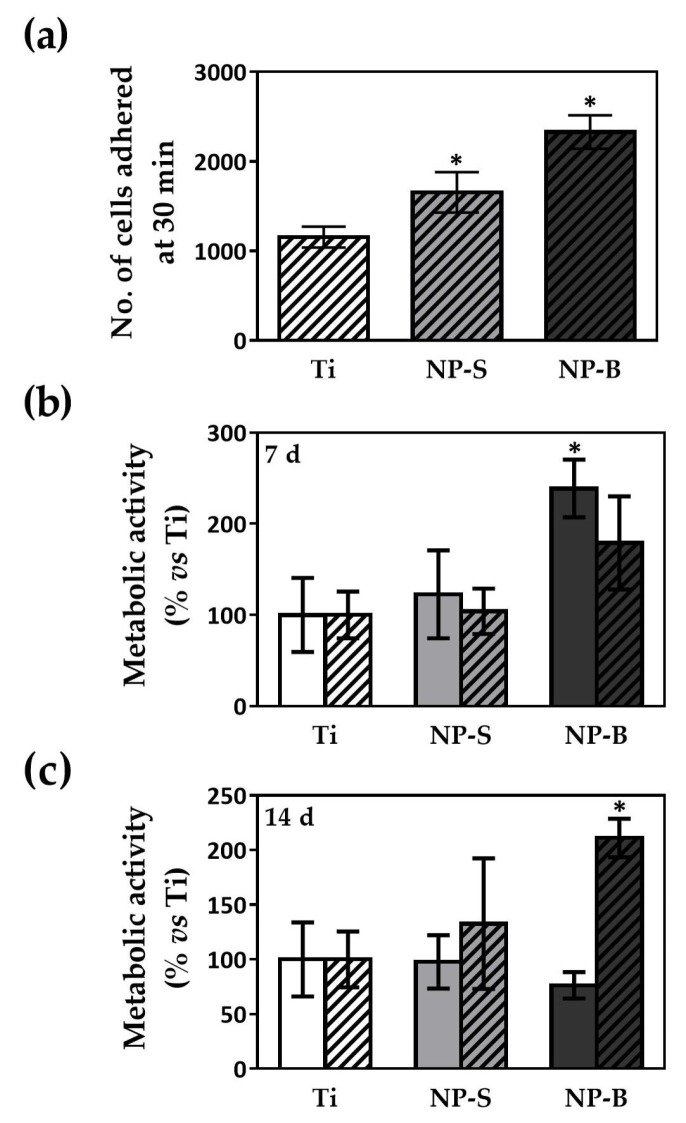
**(a)** Cell adhesion of hGF-C on the different surfaces. Number of cells adhered to the control and modified surfaces after 30 min. Data represent the mean ± SEM (*n* = 6 for donor hGF-B). Differences between groups were assessed by ANOVA and post-hoc LSD test: * *p* < 0.05 versus Ti. (**b**) Metabolic activity of hGF-A (plain) and hGF-C (striped) at day 7 and (**c**) 14 of cell culture onto different surfaces. Data are expressed as percentage of Ti control for each day, which was set to 100%. Values represent the mean ± SEM (*n* = 6). Significant differences were assessed by ANOVA and post-hoc LSD test: * *p* ≤ 0.05 versus Ti.

The nanostructuring of implant surfaces provides a mechanism to encourage and direct cell adhesion to the implant surface [27], providing an effective substrate for cell contact and proliferation [12,38]. As illustrated in Figure 3a, cell adhesion after 30 min was increased significantly by surface nanostructuration compared to the Ti control, and showed a tendency to better adhesion for higher porous diameters, although significance was only found compared to the Ti control.

Cell metabolic activity at days 7 and 14 were also evaluated (Figure 3b,c). When comparing different surfaces with Ti, results showed a different response depending on the hGF donor. Donor B showed increased metabolic activity when seeded on the NP-B group at day 14. In contrast, donor A seeded on the NP-B group showed higher metabolic activity at day 7, while no differences were found at day 14. According to previous studies, TiO_2_ nanotubular surfaces with a tube diameter of ∼120 nm improved adhesion and proliferation of hGFs [1]. However, others showed better results with diameters lower than 100 nm, with 15 nm being the best pore size [8,31]. All in all, there are some controversial discussions because there is no consensus about the optimal geometric parameters of nanostructures [39], and although many studies of different pore size have been carried out, there is an absence of comparable studies [8]. Moreover, it has been suggested that various cell types respond differently to similar nanostructures [19], and here we show that differences are even found among different donors of the same cell type.

Thus, our results show that the NP-B surface is the one inducing a better cell adhesion and metabolic activity over time, and topographically we have proved that it differs from NP-S and the Ti control on the surface area, in agreement with other studies that have demonstrated a relation between surface roughness, cell proliferation, and cell adhesion [40,41]. Previously, generation of nanotopographies and surface roughness have been used to improve osteoblast cell attachment and osseointegration of oral dental implants [42]. In the present study, we have demonstrated that formation of TiO_2_ nanoporous structures could also represent a technique for improvement of the initial attachment and spreading of the cells on the abutment implant surface for a better soft tissue attachment.

**Figure 4:**
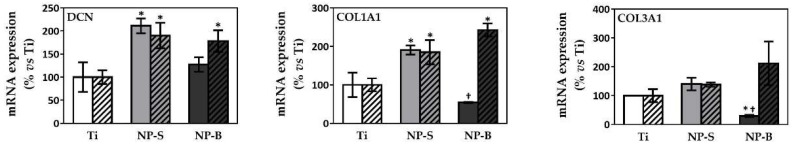
mRNA levels of gingival fibroblast differentiation markers after 14 d of cell culture. Plain bars correspond to donor hGF-A, and striped bars correspond to donor hGF-B. Data represents fold changes of target genes normalized to reference genes (*Gapdh* and *β-Actin*), expressed as percentage of control, which was set to 100%. Values represent the mean ± SEM (*n* = 3) for each donor. Differences between groups were assessed by ANOVA and post-hoc LSD test: * *p* < 0.05 versus Ti; ^†^ *p* < 0.05 versus NP-S.

**Figure 5:**
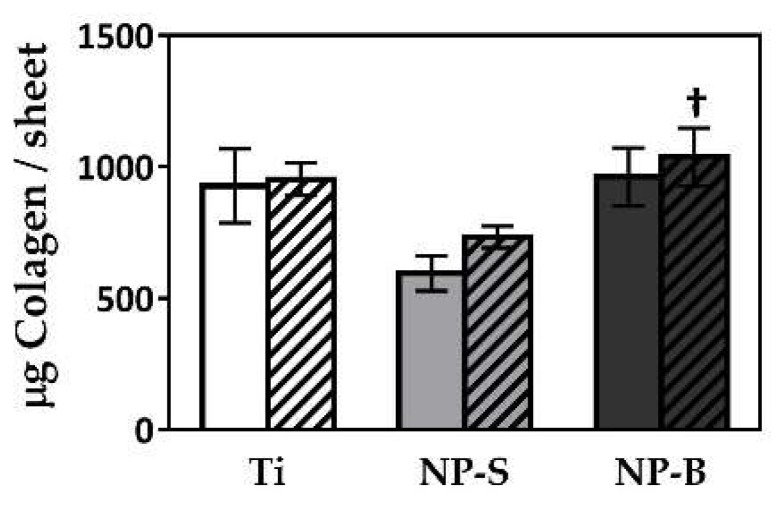
Total collagen content after 14 days of cell culture. Plain bars correspond to donor hGF-A, and striped bars correspond to donor hGF-B. Values represent the mean ± SEM (*n* = 3) for each donor. Significant differences were assessed by ANOVA and post-hoc LSD test: ^†^ *p* < 0.05 versus NP-S.

We also analyzed the gene expression levels of different genes related to the extracellular matrix (ECM), and the total amount of collagen deposition after 14 days of cell culture (Figure 4 and Figure 5). We wanted to evaluate the effect of nanoporous structures in promoting collagen rich cell culture, a key factor for ECM synthesis, and consequently an effective connective tissue after installation of a dental implant [43].

As shown in Figure 4, a differential response to the surface depending on the cell donor was also found. For donor B we observed increased expression levels of the ECM components *COL1A1*, *COL3A1*, and *DCN (Decorin)* when seeded onto the nanostructured surfaces, showing a tendency to higher expression levels while increasing the nanoporous diameter. While for donor A, we observed a better response to NP-S surfaces, and a decrease in *COL1A1* and *COL3A1* mRNA expression levels when seeded onto NP-B surfaces, and the same tendency was found for *DCN*. Differences between donors could be a consequence of the difference in their age (20 years apart) and sex. In fact, the effects of age and sex differences on the collagen turnover profile have been reported, showing the importance to address both age and sex when interpreting ECM data [44].

However, we found higher collagen deposition in cells cultured onto NP-B surfaces for both donors, although statistical significance was only achieved for donor B. One interpretation of differences in results in gene expression and collagen deposition is the fact that the extracellular collagen results from the total accumulation during the whole cell culture period, meanwhile gene expression levels were just those corresponding to the levels found at day 14 of cell culture. Thus, it may be that gene expression presents a changeable pattern during cell culture, and that the gene expression levels found at day 14 are not equal to final extracellular collagen deposition.

## 3. Materials and Methods

### 3.1. Preparation of Nanoporous Layers on Ti Foil

Highly ordered TiO_2_ nanoporous structures were grown on Ti foils with a two-step electrochemical anodization assay. Prior to the anodization process, Ti foils (99.7% purity, 0.127 mm thick, Sigma-Aldrich, St. Louis, MO, USA) pre-cut in to 0.7 cm × 8.5 cm pieces, were degreased by sonicating in acetone, ethanol, and distilled water for five minutes each in an ultrasonic bath (Branson 5510, Sigma-Aldrich). Finally, the samples were dried under a nitrogen flow.

The anodizations were conducted in a two-electrode cell with a potentiostat instrument (Metrohm Autolab BV, Utrecht, The Netherlands). Ti foil was used as an anode, and platinum electrode as a cathode (Metrohm). The electrodes were kept parallel and were submerged into the electrolyte solution in a Teflon beaker (Sigma-Aldrich). Table S1 (Supplementary Material) shows the different anodization conditions and protocols tested in order to achieve nanostructures with different porous diameters. To achieve the nanostructures further used in this study, an organic electrolyte containing 0.1 M NH_4_F (Sigma-Aldrich), 1 M H_2_O, and ethyleneglycol 99.5% pure (Sigma-Aldrich), previously aged for six and a half hours, was used as the anodizing electrolyte.

Experiments were conducted at room temperature under agitation. After a first anodization, the created oxide films were removed by mechanical detachment using Scotch^®^ Magic^TM^ adhesive tape (3 M, Cergy-Pontoise, France), and the Ti foil was assembled back with the electrolytic cell for a second anodization. Table 2 shows the conditions of voltage and the interspace between electrodes used to obtain the different pore diameter nanoporous structures. Conditions of voltage and time were modified and monitored with Nova 2.0 software (Metrohm). The samples were rinsed with ethanol, dried under a nitrogen flow, cut using scissors in to 1 × 0.7 pieces, and disinfected with dry heat (90 °C, 30 min) before cell seeding.

### 3.2. Characterization of Ti Nanopore Arrays

#### 3.2.1. SEM

For the structure and morphology characterization of nanoporous layers, a scanning electron microscope was used to acquire images (SEM, HITACHI S-3400N, Hitachi High-Technologies Europe, Krefeld, Germany), applying secondary electrons, low vacuum conditions, and 15 kV of voltage. The samples were sputtered with gold prior to analysis. The pore diameter (nm) was calculated using ImageJ software (v1.51 k, Rasband, W.S., ImageJ, US National Institutes of Health, Bethesda, MD, USA).

#### 3.2.2. AFM

Surface roughness of the nanopores was examined with atomic force microscopy (AFM, VECCO model multicode, VECCO, Plainview, Oyster Bay, NY, USA) in air tapping mode, with a scan size of 5 µm, in combination with HQ: NSC35/Al probes (Mikromasch, Lady’s Island, SC, USA) with a nominal spring constant of 16 N/m, and resonant frequency of 300 kHz. Topographical analysis was performed by importing the resulting AFM data files into Nanoscope software (v5.10, VEECO) and selecting the roughness tool. 

#### 3.2.3. Contact Angle or Surface Wettability

Surface wettability was evaluated using a static sessile water-drop method. The experiment was performed using 2 μL of ultrapure water (wetting agent). Images were taken using a Nikon D3300 (AF-P DX 18–55 mm lens, Tokyo, Japan). The contact angle was calculated using ImageJ software (Rasband, W.S., ImageJ, US National Institutes of Health, Bethesda, MD, USA).

#### 3.2.4. Nanoparticle Release

To measure nanoparticle release, the different nanoporous samples and the Ti control were incubated at 37 °C with ultrapure water for seven days. NP suspensions were analyzed by DLS (Dynamic light scattering) using Zetasizer ZS90 (Malvern Panalytical Ltd., Malvern, UK). The nanoparticle concentration was analyzed by NTA (Nanoparticle Tracking Analysis) using PMX 120 Scanning ZetaView^®^ (Particle Metrix Inc., Mebane, NC, USA). Experiments were run in duplicate for each group. Nanoparticle concentration was measured only for those samples that presented nanoparticle release, as measured by DLS.

### 3.3. Cell Culture

Primary human gingival fibroblasts (hGF) from two different donors were purchased from Provitro (GmbH, Berlin, Germany): hGF-A (27 years, female, Caucasian, 313X100401) and hGF-B (47 years, male, Caucasian, 323X070501). Provitro assures that cells were obtained ethically and legally, and that all donors provided written informed consent.

Cells were routinely cultured at 37 °C and 5% CO_2_ in DMEM (Dulbecco’s Modified Eagle’s medium) GlutaMAX low glucose medium (Life Technologies, Carlsbad, CA, USA), supplemented with 10% (*v*/*v*) fetal calf serum (Biosera, Boussens, France), 100 µg/mL penicillin, and 100 µg/mL streptomicin (Biowest, Nuaille, France). The medium was also supplemented with 50 µg/mL ascorbic acid (Sigma-Aldrich) and refreshed twice a week. Experiments were performed with hGF between passages nine (hGF-A) and seven (hGF-B) after isolation.

Cells were seeded at a density of 7.0 × 10^3^ cells/well, using the flexiPERM^®^ micro12 system (growth area 0.3 cm²) (Sarstedt, Nümbrecht, Germany) on the different nanoporous and Ti control groups.

### 3.4. Cell Cytotoxicity

To estimate cytotoxicity of Ti nanoporous samples, the presence of lactate dehydrogenase (LDH) in culture media after 48 h of cell incubation onto the different surfaces was used as an index of cell death. Following the manufacturer’s instructions (Cytotoxicity Detection kit, Roche Diagnostics, Mannheim, Germany), LDH activity was determined spectrophotometrically after 30 min of incubation at room temperature (RT) of 50 µL of culture media and 50 µL of the reaction mixture, by measuring the oxidation of nicotinamide adenine dinucleotide (NADH) at 490 nm in the presence of pyruvate.

The results were presented relative to the LDH activity of the media of cells seeded on tissue culture plastic (TCP) without treatment (low control, 0% of cell death), and on cells grown on TCP treated with 1% Triton X-100 (high control, 100% of death), using the following equation: Cytotoxicity (%) = (exp.value-low control)/(high control-low control) × 100. Experiment was run in six sample replicates (*n* = 6) for each group and donor.

### 3.5. Cell Adhesion and Metabolic Activity

Cell adhesion and metabolic activity were quantified using PrestoBlue^®^ (ThermoFisher, Waltham, Massachusetts, USA), a live-cell resazurin-based viability reagent (Life Technologies, Carlsbad, CA, USA). To determine cell adhesion, six sample replicates of one donor (hGF-B) (*n* = 6) were used. Thirty minutes after seeding, the medium was aspirated, cells were washed twice with PBS (Phosphate buffered saline), and 100 µL of fresh culture medium were added to each well with 10 µL of PrestoBlue. After an overnight incubation, the absorbance of the medium was read at 570 and 600 nm. A standard was grown in the same conditions in parallel to the samples.

Metabolic activity was determined at days 7 and 14 of hGF culture on the surfaces, using PrestoBlue reagent following the manufacturer’s protocol. The results were presented relative to the Ti control.

### 3.6. Gene Expression by Real-Time Polymerase Chain Reaction (RT-PCR)

Total RNA was isolated from the 14 days cell culture using Tripure^®^ reagent (Roche Diagnostics, Mannheim, Germany) according to the manufacturer’s protocol, and quantified at 260 nm using a Nanodrop spectrophotometer (NanoDrop Technologies, Wilmington, DE, USA).

For reverse-transcribe to cDNA, a High Capacity RNA to cDNA kit (Applied Biosystems, Foster City, CA, UA) was used, using the same amount of RNA (140 ng) for all the samples. Each cDNA was diluted, and aliquots were frozen (−20 °C) until the RT-PCRs were carried out.

Real-time PCR was performed in the Lightcycler 480^®^ system using SYBR green detection (Roche Diagnostics). It was performed for two reference genes and five target genes. The primer sequences used are detailed in Table 3.

Each reaction contained 7 μL of master mix (Lightcycler 480 SYBR Green I Master, Roche Diagnostics), the sense and the antisense specific primers (0.5 μM), and cDNA sample (3 μL), in a final volume of 10 μL. The amplification program consisted of a preincubation step for denaturation of the template cDNA (5 min, 95 °C), followed by 45 cycles consisting of a denaturation step (10 s, 95 °C), an annealing step (10 s, 60 °C), and an extension step (10 s, 72 °C). After each cycle, fluorescence was measured at 72 °C. A negative control without cDNA template was run in each assay [45].

To allow relative quantification after PCR, standard curves were constructed from standard reactions for each of the target and reference genes. The crossing point readings for each of the unknown samples were used to calculate the amount of either the target or the reference relative to a standard curve, using the Second Derivative Maximum Method provided by the LightCycler 480 analysis software version 1.5 (Roche Diagnostics, Mannheim, Germany). All samples were normalized by the mean of the expression levels of the reference genes [46].

### 3.7. Collagen Quantification

After 14 days of cell culture, cells were washed with H_2_O, dried for 1 h at RT, followed by incubation for 1 h at −80 °C. Cells were then incubated overnight at 37 °C in a humidified atmosphere, followed by 24 h at 37 °C in a dry atmosphere. Collagen was stained with 0.1% Sirius Red F3BA (Sigma-Aldrich) in saturated picric acid (Sigma-Aldrich) for 1 h at RT. Unbounded dye was removed with 10 mM HCl washes, and dye was solubilized with 0.1 M NaOH. Absorbance was measured at 540 nm. Readings were compared with a calf-skin collagen standard (Calf skin type I Collagen, Sigma-Aldrich) included in the assay. Experiment was run in triplicate (*n* = 3) for each group and donor.

### 3.8. Statistical Analysis

All data are presented as mean values ± standard error of the mean (SEM). The Kolmogorov–Smirnov test was done to assume parametric or non-parametric distributions for the normality tests. Differences between groups were assessed, depending on their normal distribution, by the Mann−Whitney U test or the two-way analysis of variance (ANOVA) test, followed by post-hoc pairwise comparisons using the LSD test. SPSS software (version 24.0, Chicago, IL, USA) and GraphPad Prism (version 7, La Jolla, CA, USA) were used. Results were considered statistically significant at *p* values < 0.05.

## 4. Conclusions

The present work shows the potential of anodic oxidation of Ti surfaces to induce an improved cell response, which could be attributed to the improvement of surface properties. We establish the electrochemical condition to achieve two different pore diameters, by controlling the applied potential and the interspacing of the electrode, showing how these two conditions determine the pore diameter. Larger porous diameters were obtained by increasing the voltage used during anodization. To obtain the smallest diameter, apart from lowering the voltage, a lower interelectrode spacing was needed. Our results show quite different topographical characteristics on surface properties—reflected on surface area—for the bigger nanoporous structures of ~74 nm compared to nanoporous structures of ~48 nm or the Ti control.

These changes of surface properties are reflected in the improvement of gingival fibroblast response for nanoporous structures of ~74 nm compared to unstructured Ti control surfaces or nanoporous surfaces with a smaller ~48 nm porous structure. With the presence of nanoporous surfaces we have stimulated cell growth and proliferation, obtaining the best results for the bigger pore diameter of ~74 nm, probably due to the higher surface area and number of peaks. Therefore, we found the optimal pore diameter for the improvement of human gingival fibroblast response, although these effects had a significant dependence on the cell donor, probably affected by the age and sex of the donors.

Thus, although further work is needed, we believe that the possibility of diameter control and surface modification with just two simple parameters holds great potential for clinical applications, having an impact in the improvement of soft tissue integration, and increasing dental implant efficacy.

## Figures and Tables

**Table 1:** Summary of TiO_2_ nanoporous and Ti control surface characteristics.

Parameters	Ti Control	NP-S	NP-B
Pore diameter (nm) ^1^	(-)	48.2 ± 1.2	74.0 ± 3.3 ^†^
R_a_ (nm)	51.7 ± 5.71	54.7 ± 1.4	41.6 ± 5.5
R_skw_ (-)	0.123 ± 0.337	0.069 ± 0.162	−0.310 ± 0.196
R_kur_ (-)	3.62 ± 0.39	2.62 ± 0.15 *	2.99 ± 0.15
R_pc_ (-)	29.8 ± 5.0	22.0 ± 4.0	156 ± 34 * ^†^
Srf. Area (µm^2^)	26.4 ± 0.2	26.4 ± 0.5	30.4 ± 0.4 * ^†^
R_sa_ (%)	5.68 ± 0.86	5.41 ± 0.21	21.6 ± 1.6 * ^†^
Contact angle (°)	53.2 ± 2.5	78.6 ± 2.2 *	65.5 ± 5.8 ^†^

^1^ Pore diameter results represent the mean ± S.E.M (Standard Error of the Mean), with *n* = 120 of each group. Absence of pores (-), Average surface roughness (R_a_), Skewness (R_skw_), Kurtosis (R_kur_), peak counts (R_pc_), surface area (Srf. Area), and surface area difference (R_sa_). Results represent the mean ± SEM, *n* = 2 for roughness measurements and *n* = 4 for contact angle measurements. Differences were determined by ANOVA (Analysis of Variance), using a post-hoc LSD (Least significant difference) test; * *p* < 0.05 versus Ti: ^†^ *p* < 0.05 versus NP-S.

**Table 2:** Conditions for the two-step anodizing process carried out. Voltage conditions applied and the interspace between the electrodes used during anodization.

TiO_2_ Structure	First Anodizing Step 30 min	Second Anodizing Step 10 min	Interspace
NP-S	35 V ^1^	1 V	2.5 cm
NP-B	60 V	60 V	5 cm

^1^ V (voltage).

**Table 3:** Sequence of sense (S) and antisense (A) primers used in the real-time polymerase chain reaction (PCR) of reference and target genes. Base pairs (bp).

Gene	Primer Sequence (5′–3′)	Product Size (bp)	GenBank ID
Collagen I α1 (*COL1A1*)	S: AGAGCATGACCGATGGATTC A: TTCTTGAGGTTGCCAGTC	122	NM_000088.3
Collagen III α1 (*COL3A1*)	S: GGCCTACTGGGCCTGGTGGT A: CCACGTTCACCAGGGGCACC	190	NM_000090.3
Decorin (*DCN*)	S: ATCTCAGCTTTGAGGGCTCC A: GCCTCTCTGTTGAAACGGTC	146	NM_001920.3
Glyceraldehyde-3-Phosphate Dehydrogenase (*GAPDH*)	S: TGCACCACCAACTGCTTAGC A: AAGGGACTTCCTGTAACAA	87	NM_002046.3
β-Actin (*ACTBL2*)	S: CTGGAACGGTGAAGGTGACA A: AAGGGACTTCCTGTAACAA	140	NM_001101.3

## Supplementary Materials

Supplementary materials can be found at http://www.mdpi.com/1422-0067/19/10/2881/s1.

## Abbreviations

hGF	Human gingival fibroblasts
MSCs	Mesenchymal stem cells
Ti	Titanium
TiO_2_	Titanium dioxide
NP	Nanopore
SEM	Scanning electronic microscope
R_a_	Average surface roughness
R_q_	Root Mean Square (RMS) roughness
R_max_	Maximum surface roughness
R_skw_	Skewness
R_kur_	Kurtosis
R_pc_	Peak counts
R_sa_	Surface Area difference
AFM	Atomic force microscope
LDH	Lactic dehydrogenase
NADH	Nicotinamide adenine dinucleotide
DLS	Dynamic light scattering
S.E.M.	Standard Error of the Mean
ANOVA	Analysis of variance
LSD	Least significant difference
ECM	Extracellular matrix
GAPDH	Glyceraldehyde-3-phosphate dehydrogenase
COL1A1	α-1 type I collagen
COL3A1	α-1 type III collagen
DCN	Decorin
TCP	Tissue culture plastic
