# Supplementary material for: Tuning Nanopore Diameter of Titanium Surfaces to Improve Human Gingival Fibroblast Response
Source: Int J Mol Sci. 2018 Sep 22;19(10):2881. doi: 10.3390/ijms19102881 (PMC6213077; doi:10.3390/ijms19102881)
Supplement: Supplementary file 1 [file ijms-19-02881-s001.pdf]

**Supplementary Table S1.** Different anodization conditions and protocols tested in order to achieve nanostructures with different porous diameters. All protocols were conducted with the electrolyte aged for 400–900 min and a peeling step with Scotch Magic® between the two anodizations.

| Electrolyte | Electrodes Interspace | Anodization Conditions | SEM | Comments |
|---|---|---|---|---|
| 0.14 M $NH_4H$<br>2.78 M $H_2O$<br>EG | 5 cm | 35 V 30 min<br>10 V 30 min | 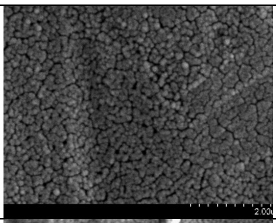 | No nanostructure was obtained. |
| 0.1 M $NH_4H$<br>1.67 M $H_2O$<br>EG | 5 cm | 35 V 30 min<br>10 V 30 min | 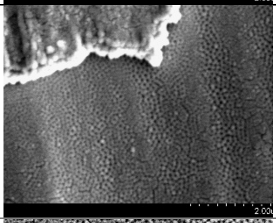 | Heterogenous surface with two layers. |
| 0.1 M $NH_4H$<br>1 M $H_2O$<br>EG | 5 cm | 60 V 30 min<br>10 V 2.5 h | 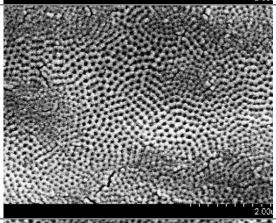 | Heterogenous nanoporous surface obtained. |
| 0.1 M $NH_4H$<br>1 M $H_2O$<br>EG | 5 cm | 60 V 30 min<br>10 V 1 h | 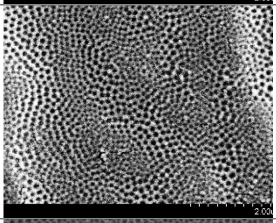 | With a shorter reaction time, a heterogenous nanoporous surface was also obtained. |
| 0.1 M $NH_4F$<br>1 M $H_2O$<br>EG | 5 cm | 60 V 30min<br>60 V 10 min | 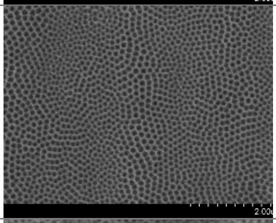 | With a shorter reaction time and higher voltage, a homogeneous nanoporous surface obtained. **Conditions used in the study for NP-B.** |
| 0.1 M $NH_4F$<br>1 M $H_2O$<br>EG | 5 cm | 60 V 30min<br>10 V 10 min | 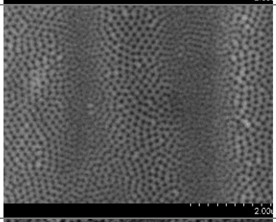 | Lower voltage. Homogeneous nanoporous surface obtained. Smaller pore diameter |
| 0.1 M $NH_4F$<br>1 M $H_2O$<br>EG | 2.5 cm | 60 V 30min<br>1 V 10 min | 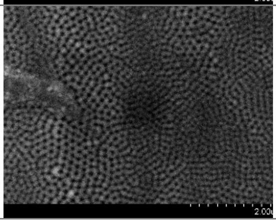 | Lower voltage and inter-electrode spacing. Heterogenous nanoporous surface obtained. |

| | | | | |
|---|---|---|---|---|
| 0.1 M $NH_4H$<br>1 M $H_2O$<br>EG | 5 cm | 35 V 30 min<br>3 V 1 h | 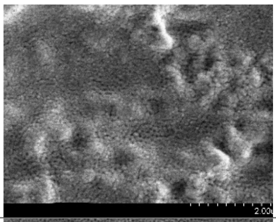 | No nanostructure was obtained. |
| 0.1 M $NH_4H$<br>1 M $H_2O$<br>EG | 5 cm | 35 V 30 min<br>1 V 1 h | 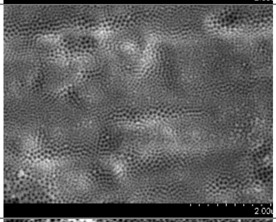 | Lower voltage.<br>Heterogenous nanoporous surface obtained |
| 0.1 M $NH_4F$<br>1 M $H_2O$<br>EG | 5 cm | 35 V 30min<br>1 V 10 min | 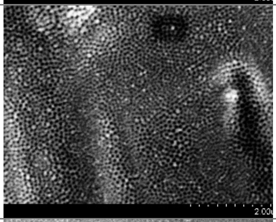 | Lower reaction time.<br>Heterogenous nanoporous surface obtained |
| 0.1 M $NH_4F$<br>1 M $H_2O$<br>EG | 2.5 cm | 35 V 30min<br>1 V 10 min | 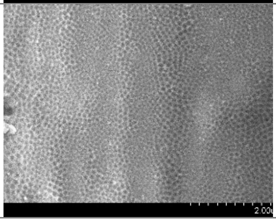 | Lower voltage and inter-electrode spacing.<br>Homogeneous nanoporous surface obtained.<br>**Conditions used in the study for NP-S.** |
